# Genetic Heterogeneity of Hepatitis C Virus in Association with Antiviral Therapy Determined by Ultra-Deep Sequencing

**DOI:** 10.1371/journal.pone.0024907

**Published:** 2011-09-22

**Authors:** Akihiro Nasu, Hiroyuki Marusawa, Yoshihide Ueda, Norihiro Nishijima, Ken Takahashi, Yukio Osaki, Yukitaka Yamashita, Tetsuro Inokuma, Takashi Tamada, Takeshi Fujiwara, Fumiaki Sato, Kazuharu Shimizu, Tsutomu Chiba

**Affiliations:** 1 Department of Gastroenterology and Hepatology, Graduate School of Medicine, Kyoto University, Kyoto, Japan; 2 Department of Gastroenterology and Hepatology, Osaka Red Cross Hospital, Osaka, Japan; 3 Department of Gastroenterology and Hepatology, Wakayama Red Cross Hospital, Wakayama, Japan; 4 Department of Gastroenterology, Kobe City Medical Center General Hospital, Kobe, Japan; 5 Department of Gastroenterology and Hepatology Takatsuki Red Cross Hospital, Osaka, Japan; 6 Department of Nanobio Drug Discovery, Graduate School of Pharmaceutical Sciences, Kyoto University, Kyoto, Japan; Veterans Affairs Medical Center (111D), United States of America

## Abstract

**Background and Aims:**

The hepatitis C virus (HCV) invariably shows wide heterogeneity in infected patients, referred to as a quasispecies population. Massive amounts of genetic information due to the abundance of HCV variants could be an obstacle to evaluate the viral genetic heterogeneity in detail.

**Methods:**

Using a newly developed massive-parallel ultra-deep sequencing technique, we investigated the viral genetic heterogeneity in 27 chronic hepatitis C patients receiving peg-interferon (IFN) α2b plus ribavirin therapy.

**Results:**

Ultra-deep sequencing determined a total of more than 10 million nucleotides of the HCV genome, corresponding to a mean of more than 1000 clones in each specimen, and unveiled extremely high genetic heterogeneity in the genotype 1b HCV population. There was no significant difference in the level of viral complexity between immediate virologic responders and non-responders at baseline (p = 0.39). Immediate virologic responders (n = 8) showed a significant reduction in the genetic complexity spanning all the viral genetic regions at the early phase of IFN administration (p = 0.037). In contrast, non-virologic responders (n = 8) showed no significant changes in the level of viral quasispecies (p = 0.12), indicating that very few viral clones are sensitive to IFN treatment. We also demonstrated that clones resistant to direct-acting antivirals for HCV, such as viral protease and polymerase inhibitors, preexist with various abundances in all 27 treatment-naïve patients, suggesting the risk of the development of drug resistance against these agents.

**Conclusion:**

Use of the ultra-deep sequencing technology revealed massive genetic heterogeneity of HCV, which has important implications regarding the treatment response and outcome of antiviral therapy.

## Introduction

Hepatitis C virus (HCV) is classified as a member of the Flaviviridae family [1] and has an approximately 9.6-kb single-stranded RNA genome. This RNA genome encodes a large precursor polyprotein, which is cleaved by viral and host proteases to generate at least 10 functional viral proteins; core, envelope (E)-1, E2, p7, nonstructural protein (NS)-2, NS3, NS4A, NS4B, NS5A, and NS5B [2], [3]. A strong characteristic of HCV infection is its significant genetic diversity, the consequence of the absence of proofreading activity in RNA-dependent RNA polymerase [4], and the high level of viral replication during its life cycle [5]. The mean frequency of nucleotide alterations occurring in HCV RNA is calculated to be between 1.4×10^3^ and 1.9×10^3^ substitutions per nucleotide per year [6], [7]. As a result, the infecting HCV clones in each patient invariably show population diversity with a high degree of genetic heterogeneity. The collection of viruses in a population of closely related but non-identical genomes is referred to as a quasispecies [8], [9], and the dominant viral population may be evolving as a result of its viral replicative fitness and concurrent immune selection pressures that drive clonal selection.

It is reasonable to assume that the viral pathogenesis and sensitivity to treatment are affected by the generation of escape mutants through immune evasion and the modification of virulence characteristics by anti-viral treatment [10]. Thus, certain viral mutations have important implications for the pathogenesis of the viral disease and the sensitivity to antiviral therapy. Several studies have attempted to associate genetic heterogeneity or number of mutations with pathogenesis and treatment outcome. However, the abundant diversity and complexity of the chronically-infected HCV has been an obstacle to evaluate the viral genetic heterogeneity in detail. In this respect, the recent introduction of ultra-deep sequencing technology, capable of producing millions of DNA sequence reads in a single run, is rapidly changing the landscape of genome research [11], [12]. One application of ultra-deep sequencing was the identification of rare minority drug resistant clones of human immunodeficiency virus, which are not detectable by standard sequencing techniques [13]–[15]. Moreover, the recent study using 454/Roche pyrosequencing technology clarified the transmission bottlenecks by measuring the population structure within patients with HCV infection [16].

In this study, we used for the first time ultra-deep sequencing with Illumina Genome Analyzer II (Illumina, San Diego, CA) and determined the pictures of viral quasispecies of genotype 1b HCV in patients receiving peg-interferon (IFN) α2b plus ribavirin (RBV) to clarify the significance of the viral genetic complexity in the pathophysiology of HCV infection and the treatment outcome of the current IFN-based therapy for HCV-infected patients. Because our main objective was to determine whether the HCV sequence variation itself is responsible for the sensitivity or resistance to antiviral therapy, we compared the composition of the HCV population complexity 1 week after IFN administration in patients who showed a prompt decrease in HCV viremia with those in whom there was no reduction in the serum HCV RNA levels after the initiation of IFN treatment. We also examined the prevalence of drug-resistant mutations to direct-acting antivirals (DAAs) for HCV in treatment-naïve HCV-infected patients, based on the fact that drug-resistant mutations already exist in treatment-naïve patients with various pathogenic virus infections, such as human immunodeficiency viruses [14], [17].

## Results

### Validation of multiplex ultra-deep sequencing of the HCV genome

We performed a massive parallel ultra-deep sequencing run on the Illumina Genome Analyzer II platform using multiplex tagging methods. First, we conducted a control experiment to validate the efficacy and error rates in ultra-deep sequencing of the viral genome. For this purpose, we used a plasmid encoding full-length HCV [18] as a template and determined the plasmid-derived whole HCV sequence. The ultra-deep sequencing platform provided us the full-length HCV genome information derived from the plasmids with a mean coverage of 1674.3 at each nucleotide site (Table 1). Errors comprised insertions (1.0%), deletions (4.2%), and nucleotide mismatches (94.8%) and the overall error rates by multiplex ultra-deep sequencing were determined to be a mean of 0.0010 per bp. Next we confirmed that the high-fidelity PCR amplification with HCV-specific primer sets followed by multiplex ultra-deep sequencing resulted in no significant increase in the error rates in the viral sequencing data (ranging from 0.0012 to 0.0013 per bp; per-nucleotide error rate, 0.12%–0.13%).

**Table 1:** Error frequency of ultra-deep sequencing for the plasmid encoding full-genome HCV sequence.

	PCR amplification	
	(−)*	(+)*
Total read nucleotides	15,118,929	24,158,372
Mean coverage	1674.3	5562.6
Type of errors		
mismatches	14,629 (94.8%)	26,243 (88.6%)
deletions	640 (4.2%)	2510 (8.5%)
insertions	147 (1.0%)	859 (2.9%)
Overall error rate (%)	0.102	0.123

*(−); Ultra-deep sequencing of HCV encoding plasmid

(+); Ultra-deep sequencing of PCR-amplified HCV encoding plasmid.

To estimate the accuracy of detecting nucleotide alterations using reads filtered by average base quality and mapping quality, we introduced the plasmid with single point mutations within the wild-type viral sequences with the ratio of 1∶99 and 1∶999 and assessed the sensitivity and accuracy of quantification with the high-fidelity PCR amplification followed by multiplex ultra-deep sequencing. Duplicate control experiments revealed that mutations present at an input ratio of 0.10% ranged between 0.09 and 0.19%, and the results could be reproducibly quantified (data not shown). Based on these results, we picked up the low abundant mutations that presented at frequency of more than 0.20% among the total viral clones, a level that could rule out putative errors caused by massively-parallel sequencing, in the current platform used in this study.

### Large heterogeneity of viral clones in HCV-infected patients

HCV infection comprises a heterogeneous mixture of viral clones with various mutations. To clarify the landscape of HCV heterogeneity as a quasispecies, we determined the viral full-genome sequences derived from 27 HCV-infected patients by multiplex ultra-deep sequencing and compared the results with those obtained by the direct population Sanger sequencing method. All sequence reads by multiplex ultra-deep sequencing have been deposit in DNA Data Bank of Japan Sequence Read Archive (http://www.ddbj.nig.ac.jp/index-e.html) under accession number DRA000366.

HCV nucleotide sequence reads by ultra-deep sequencing were aligned to the consensus viral sequences in the same serum specimen that were determined by direct population Sanger sequencing. A mean number of 1705-fold coverage on average was achieved at each nucleotide site of the HCV sequences in each specimen. The average frequencies of altered sequences detected in each viral genomic region are summarized in Table 2. Compared with the representative sequence of the population average clone, the mutation frequency was 1.04% of the total viral genomic sequences and 16.1% of the total nucleotide positions on average. Most of the genomic changes observed in viral variants were single base substitutions and unevenly distributed throughout the region of the HCV genome.

**Table 2:** Mean genetic complexity of the genotype1b HCV in chronically infected 27 patients.

Viral genomic Region	Mean number of aligned nucleotides	Mean number of mutated nucleotides	Mean coverage	Mutation frequency (%)	MeanShannon entropy
Core	779,839	5027	1361	0.61	0.045926
E1	739,220	7902	1360	0.99	0.064884
E2	1,382,907	19,724	1265	1.37	0.088584
p7	217,000	3237	1148	1.44	0.075829
NS2	673,579	8702	1073	1.19	0.075333
NS3	4,958,188	52,204	2619	0.93	0.060767
NS4A	427,677	5604	2640	1.32	0.072217
NS4B	1,209,000	17,485	1544	1.26	0.063190
NS5A	2,034,626	28,820	1518	1.28	0.067398
NS5B	2,720,417	27,449	1681	0.90	0.054805
Total	14,875,801	172,327	1705	1.04	0.062624

Among the viral genomic regions, the nucleotide sequence complexity expressed as the Shannon entropy was smallest in the core region. In contrast, the viral sequence complexity in the E2 region was highest among the HCV genomic regions and significantly greater than the average mutation frequency of the remaining HCV genome (p = 0.0026). Similarly, the ratio of the number of mutated nucleotides to the total number of nucleotides analyzed in the E2 region was significantly higher than that of the remaining HCV genome (p = 5.66×10^−6^). These findings clearly confirmed that the quasispecies complexity in E2, which contains hypervariable region1 (HVR1) and HVR2, was prominently larger than that of other viral genomic regions [19].

### Early dynamic changes of viral complexity after the administration of peg-IFNα2b plus RBV

Among 27 patients enrolled in this study, 8 showed a prompt decrease in their serum HCV RNA levels and 8 showed no significant changes 1 week after initiating treatment with peg-IFNα2b plus RBV. To clarify the changes in the viral quasispecies in response to antiviral therapy, we determined the early dynamic changes in viral complexity before and after 1 week of peg-IFNα2b plus RBV administration in these 8 immediate virologic responders and 8 non-responders. All cases were infected with genotype 1b viruses, and the clinical features, including serum HCV RNA level at baseline, did not significantly differ between immediate virologic responders and non-responders (Table 3). A mean coverage of 1798-fold and 2416-fold were mapped to each reference sequence in immediate virologic responders before and after peg-IFNα2b plus RBV administration, respectively. Similarly, a mean coverage of 1780-fold and 2461-fold were determined in non-responders before and after peg-IFNα2b plus RBV administration, respectively (Table 4 and Table S1).

**Table 3:** Characteristics of patients that showed immediate virologic response or non-response to PEG-IFNα2b plus ribavirin combination therapy.

	Immediate virologic responders	Non-responders	P-value
Age†	50.5 (45–68)	60 (55–69)	0.12
Sex (male/female)	5/3	5/3	1
Alanine aminotransaminase† (IU/l)	54 (15–198)	72 (30–143)	0.51
Total bilirubin† (mg/dl)	0.6 (0.4–1.8)	0.8 (0.4–1.4)	0.34
Platelet count† (×10^4^/mm^3^)	18.9 (7.1–27.2)	16.7 (11.6–22.5)	0.68
HCV genotype	1b	1b	
HCV viral load† (log IU/ml)			
pre-treatment	6.6 (6.2–7.5)	6.9 (6.1–7.6)	0.43
after treatment	4.6 (4.0–5.2)	6.5 (6.1–6.8)	**0.028**
Final outcome			**0.025**
sustained viral response	6	0	
Relapse	1	1	
non-response	0	6	
withdraw*	1	1	

† Values are median (range).

* The treatment was discontinued in one immediate virologic responder and one non-responder, due to the side effect of IFN and the development of liver cancer, respectively.

**Table 4:** Genetic complexity at pre-treatment and 1 week after PEG-IFNα2b plus ribavirin combination therapy in immediate virologic responders and non-responders.

	Immediate virologic responders (N = 8)		Non-responders (N = 8)	
	Pre-treatment	1 week after IFN therapy	Pre-treatment	1 week after IFN therapy
Mean number of aligned reads	263,452	356,963	256615	354,398
Mean number of aligned nucleotides	16,632,186	22,438,125	16,248,820	22,379,922
Mean coverage	1798	2416	1780	2461
Mutation frequency (%)	0.96	0.63	1.13	1.11
Shannon entropy	0.072*	0.049*	0.075**	0.066**

Wilcoxon rank sum test.

* p = 0.037.

** p = 0.12.

We then estimated the genomic complexity by calculating the Shannon entropy for each nucleotide position before and after the administration of peg-IFNα2b plus RBV (Table 4). There was no significant difference in the level of viral complexity between immediate virologic responders and non-responders at a baseline (mean Shannon entropy value 0.072 vs 0.075, p = 0.39). Immediate virologic responders, however, showed a significant reduction in the nucleotide sequence complexity after the administration of peg-IFNα2b plus RBV (mean Shannon entropy value 0.072 vs 0.049, p = 0.037), indicating that the viral quasispecies nature after the peg-IFNα2b plus RBV treatment became relatively more homogeneous than at baseline status in this group. In contrast, no significant changes in the nucleotide sequence complexity were observed in non-responder patients before and after treatment with peg-IFNα2b plus RBV (mean Shannon entropy value 0.075 vs 0.066, p = 0.12). We then examined whether specific nucleotide position might be associated with the response to peg-IFNα2b plus RBV treatment in immediate virologic responders, but complexity was not commonly shared at any specific nucleotide position that changed by more than 50% after peg-IFNα2b plus RBV administration (data not shown), indicating no association between the specific nucleotide position and the response to peg-IFNα2b plus RBV treatment.

### Elimination of minor viral clones by peg-IFNα2b plus RBV therapy

**Figure 1:**
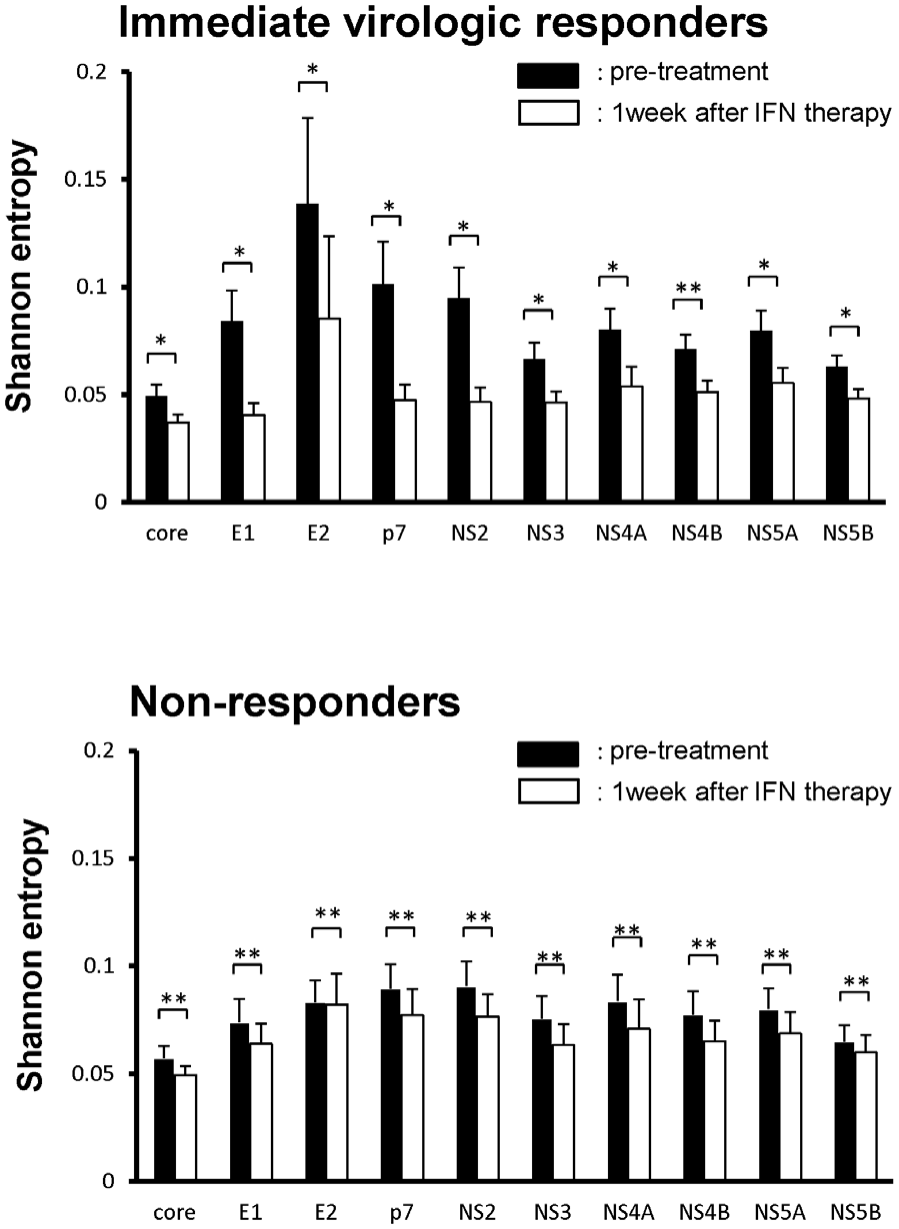
Changes in the genetic complexity of each HCV genomic region before and after the administration of peg-IFNα2b plus RBV. Shannon entropy values at baseline (black bar) and 1 week after initiation of treatment with peg-IFNα2b plus RBV (white bar) in 8 immediate virologic responders (A) and in 8 non-responders (B) are shown. * p<0.05, ** not significant. (Mean values ± SD; n = 8)

Next, we compared the nucleotide complexity in each viral genomic region of the immediate virologic responders with that of non-responders before and after peg-IFNα2b plus RBV administration (Figure 1 and Table S2). In immediate virologic responders, the peg-IFNα2b plus RBV therapy induced a significant reduction in the nucleotide sequence complexity in all viral genomic regions except NS4B. In contrast, non-responders showed no significant change in the viral sequence complexity in any viral genomic region. For example, there was no significant difference in the mean complexity in the E2 region at baseline between the immediate virologic responders and non-responders. The administration of peg-IFNα2b plus RBV significantly reduced the levels of nucleotide sequence complexity in the E2 region in all the immediate virologic responders (mean Shannon entropy value 0.139 vs 0.085, respectively. p = 0.012, Figure 1 and Table S2). In contrast, no significant changes in the sequence complexity were observed in the E2 (mean Shannon entropy value 0.083 vs 0.082, respectively. p = 0.89) regions in non-responder cases after treatment with peg-IFNα2b plus RBV.

**Figure 2:**
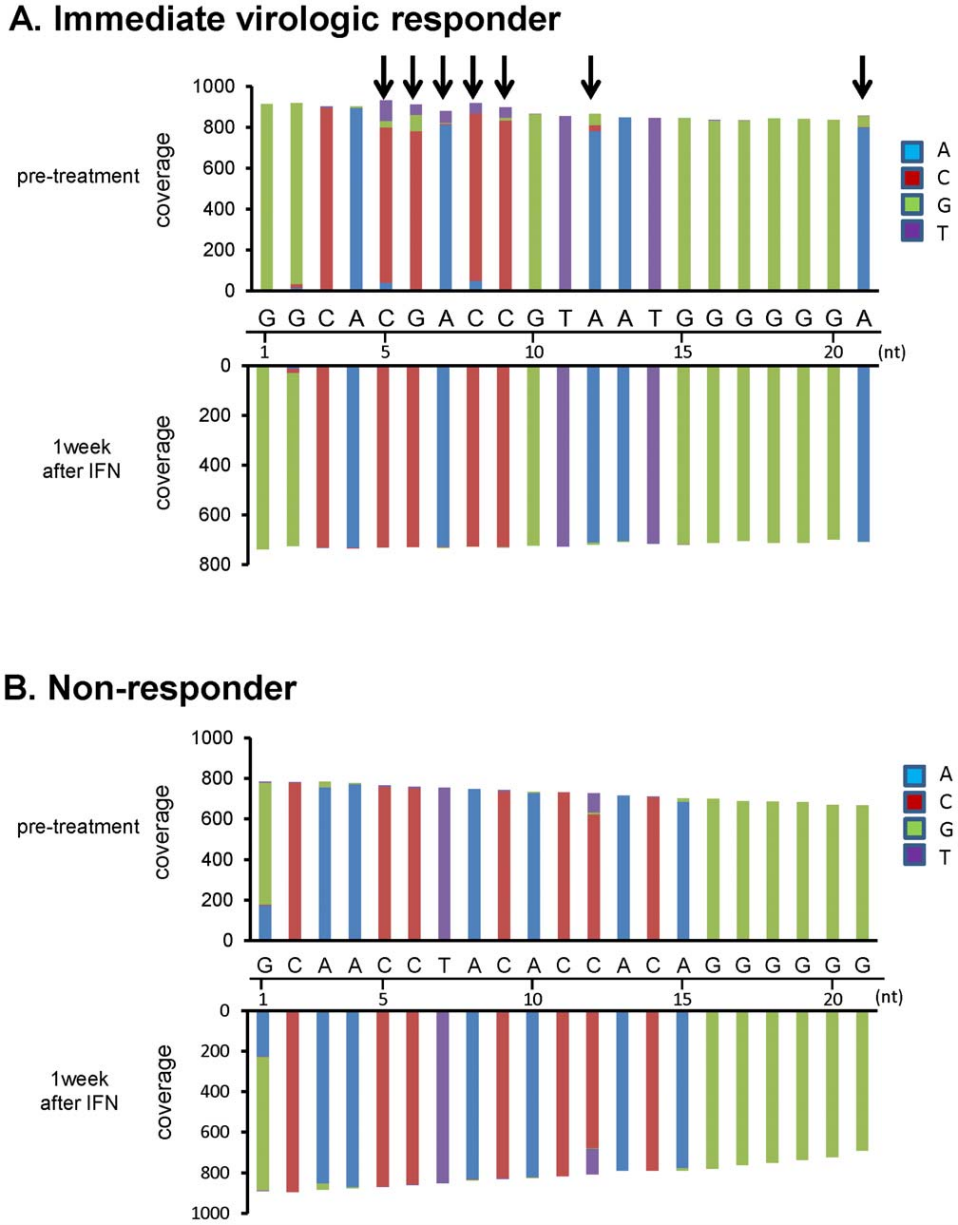
Ratio of mutated nucleotides in the HVR1 region before and after administration of peg-IFNα2b plus RBV therapy. Representative results of a immediate virologic responder (Patient#3) (**A**) and a non-responder (Patient#9) (**B**) are shown. The read numbers (coverage) at each nucleotide position of the HVR1 (from 1^st^ nucleotide to 21^st^ nucleotide in E2 region) at pre-treatment (upper graphs) and 1 week after initiating treatment with peg-IFNα2b plus RBV (lower graphs) are shown. Arrows indicate the nucleotide positions that showed the elimination of minor mutant clones after administration of peg-IFNα2b plus RBV.

To examine whether certain viral clones in non-responders showed sensitivity to IFN therapy, we investigated the sequence complexity in HVR1 in the E2 region in detail before and after peg-IFNα2b plus RBV therapy, because the HVR1 region possessed one of the highest complexities among viral genomic regions. In immediate virologic responders, the heterogeneity at each nucleotide position was reduced in response to peg-IFNα2b plus RBV administration (representative nucleotide changes are shown in Figure 2A). In contrast, the ratio of mutated clones among the total sequence reads determined at each nucleotide site in HVR1 showed no significant change before and after the administration of peg-IFNα2b plus RBV in the majority of non-responders (Figure 2B), suggesting that very few viral clones showed sensitivity to peg-IFNα2b plus RBV and were eliminated after the administration of peg-IFNα2b plus RBV.

### Detection of viral clones with drug-resistant mutations

Because none of the DAAs for HCV were approved by Japanese health coverage at the time of this study, all patients enrolled into this study were naïve to DAAs for HCV including protease and polymerase inhibitors. Thus, we determined whether the reported drug-resistant mutants exist spontaneously in nature among treatment-naïve HCV-infected patients. For this purpose, we examined the naturally prevalent mutations against HCV protease and polymerase inhibitors in the 27 patients. The drug-resistant mutations examined here included 9 mutations resistant to NS3/4 protease inhibitors, including Telaprevir, Boceprevir, TMC435350, ITMN191/R7227, MK-7009, and BI-201335, and 5 mutations resistant to NS5B polymerase inhibitors, including Filibuvir, BI-207127, and R7128 [20].

The mean number of sequence reads at the nucleotide position comprising mutations resistant to NS3/4A protease and NS5B polymerase inhibitors among the 27 cases were obtained with 1179-fold and 1972-fold coverage, respectively. Based on the detection rate of the low-level viral clones determined by the control experiments, we picked up the drug-resistant mutants that presented at a frequency of more than 0.2% among the total viral clones. Based on these criteria, at least one resistant mutation was detected in all subjects (Table 5). The mean prevalence of the 14 drug-resistant mutations ranged from 0.20% to 99.1% indicating that the proportion of resistant mutations substantially differed in each case. The T54S/A mutation resistant to Teraprevir and Boceprevir in genotype 1b HCV [21] was the most commonly detected (20 of 27 cases, 74.1%). The proportion of T54S/A mutations among the total clones ranged from 0.21% to 86.9% and thus substantially differed between cases. Other mutations resistant to the NS3/4A protease-inhibitor were detected in 16 of 27 cases (59.3%) at V55A and Q80R/K, and 12 of 27 cases (44.4%) at V36A/M. In contrast, no D168A/V/T/H mutation resistant to ITMN191/R7227, MK-7009, TMC435350, and BI-201335 was detectable. Regarding NS5B polymerase inhibitors, the V499A mutation resistant to BI-207127, was most frequently detected and 20 of 27 (74.1%) of subjects possessed the resistant-mutant clones at levels 0.20% to 99.1% at baseline. Only one case had the BI-207127-resistant P496A mutant clones and none had the R7128-resistant S282T clones. Of the 27 subjects, 16 (59.3%) harbored mutations resistant to at least four kinds of NS5B polymerase inhibitors and/or NS3/4A protease-inhibitors. Moreover, 5 subjects (18.5%) harbored resistance to 6 antiviral drugs. Notably, 3 subjects harbored resistance to 8 of 9 antiviral drugs. There was no significant association between the frequency of drug-resistant mutations and the serum viral load (r = 0.0678) (Figure S1).

**Table 5:** Prevalence of anti-HCV drug resistant mutations among the treatment-naïve patients.

Residue and Position	Drugs	Number of patients with mutated clones (%)	Frequency of the mutated clones (%)*
**Resistant mutation to NS3/4A protease inhibitor**			
T54S/A	Telaprevir Boceprevir	20/27 (74.1%)	0.49 (0.21–86.9)
V55A	Boceprevir	16/27 (59.3%)	0.4 (0.23–1.53)
Q80R/K	TMC435350	16/27 (59.3%)	0.36 (0.24–1.37)
V36A/M	Telaprevir Boceprevir	12/27 (44.4%)	0.47 (0.20–0.88)
V170A/T	Boceprevir	11/27 (40.7%)	0.52 (0.20–1.03)
A156T/V	Telaprevir	7/27 (25.9%)	0.35 (0.20–0.80)
R155K/T/Q	Telaprevir Boceprevir ITMN191/R7227 MK-7009 TMC435350 BI-201335	5/27 (18.5%)	0.42 (0.22–0.62)
A156S	Telaprevir Boceprevir	3/27 (11.15)	0.35 (0.24–0.83)
D168A/V/T/H	ITMN191/R7227 MK-7009 TMC435350 BI-201335	0/27 (0%)	
**Resistant mutation to NS5B polymerase inhibitor**			
V499A	BI-207127	20/27 (74.1%)	0.59 (0.20–99.1)
M423T/I/V	Filibuvir	12/27 (44.4%)	0.41 (0.21–1.48)
P495S/L/A/T	BI-207127	9/27 (33.3%)	0.37 (0.21–0.87)
P496A/S	BI-207127	1/27 (3.7%)	0.32
S282T	R7128	0/27 (0%)	

* Values are median (range).

These findings indicate that drug-resistant HCV variants are present in a considerable proportion among the chronically HCV-infected, DAAs-naïve patients.

## Discussion

Sequence heterogeneity, so-called quasispecies, is a common feature of RNA viruses, including HCV [22]. Previous studies of the viral genome with conventional Sanger sequencing methods revealed that HCV infection comprises a cloud of closely related sequence variants differing by as little as one nucleotide from a population average sequence [23]. A number of studies have aimed to clarify the significance of viral mutations in association with clinical features, including viral persistency and chronicity, degree of liver damage, response to treatment, and selection of mutants resistant to anti-viral therapy. The quasispecies nature of HCV, however, represents a major obstacle in determining the significance of the viral clone with specific sequence characteristics. Newly developed ultra-deep sequencing analysis allowed us to clarify the whole picture of viral quasispecies present in chronically HCV-infected patients. In the present study, ultra-deep sequencing determined a mean total of more than 10 million nucleotides of the viral genome in each specimen, representing more than 1000 clones infecting each patient, thus demonstrating the abundant genetic complexity of HCV.

It is well recognized that the HCV genome is heterogeneous at the intra-individual level [9], [10]. The current ultra-deep sequencing analyses revealed that the E2 region had the highest sequence heterogeneity, while the core region had the lowest sequence heterogeneity among the viral genomic regions encoding different functional viral proteins. More than 15% of nucleotides in the E2 region were mutated in all cases examined. These findings are consistent with previous conventional Sanger sequencing-based studies showing that HVR1 and HVR2 possess the highest sequence diversity among the HCV genomic regions [19] and that the highest values of mean Shannon entropy at the HCV 1a population level are in the E2 region [24].

Various mutations in the HCV genome are associated with the therapeutic response. For example, a number of mutations within a so-called IFNα sensitivity determining region of NS5A are closely associated with sensitivity to IFN-based anti-viral therapy [25], [26]. A recent study also showed that amino acid substitution in the HCV core region could be a useful predictor of the virologic response to peg-IFNα plus RBV combination therapy [27]. Although the findings of these studies suggested that certain mutations in the representative HCV clone could predict treatment outcome, it is unknown whether the specific viral clone comprising those mutations directly displays sensitivity or resistance to anti-viral therapy. In the present study, sequential comparison of the HCV1b genome derived at baseline and at 1 week after the administration of peg-IFNα2b plus RBV demonstrated that IFN treatment resulted in no selective decrease of the viral clones comprising the previously defined mutational changes that were associated with a response to anti-viral therapy. Moreover, immediate virologic responders showed no common baseline nucleotide alterations that are efficiently eliminated in response to the administration of peg-IFNα2b plus RBV. Thus, our data suggest that an HCV sequence variation itself at a specific single nucleotide position does not directly reflect the virologic features regarding the sensitivity to IFN therapy in each viral clone, at least at the early stage of IFN administration. In contrast, several studies have provided evidence of the pre-existence of viral strains with an inherent resistance to IFN in patients who subsequently experienced a viral breakthrough or relapse [24], [28]. Thus, there is room for further investigation to identify IFN-resistant clones by comparing the viral clones at baseline with those at the point of relapse using ultra-deep sequencing technology.

Notably, a distinct pattern of dynamic changes of HCV quasispecies was present between immediate responders and non-responders. Immediate responders showed a significant decrease of genetic complexity spanning all the viral genetic regions, resulting in a more homogeneous viral population after 1 week of peg-IFNα2b plus RBV administration. In contrast, non-responders showed no significant change in the genetic complexity in any of the HCV genomic regions. Our findings are consistent with the previous study showing that the early changes in HCV quasispecies determined by E1/E2 sequences provided prognostic information as early as the first 2 weeks after starting IFN therapy [28]. Moreover, the findings that there is no difference in the level of genetic complexity between early responders and non-responders at baseline and that almost none of the pre-existed HCV clones were eliminated in non-responder cases might suggest that the absence of sensitivity to IFN treatment in non-responders is due to host factors. Consistent with this hypothesis, recent studies revealed that host genetic variations at the IL28B gene are associated with a virologic response to peg-IFNα plus RBV combination therapy [29]-[32]. Alternatively, it is possible that a particular HCV protein of certain HCV mutants contributed to the strong inhibition of IFN-mediated anti-viral response in the liver of non-responders. Although dynamic changes in HVR1 sequences revealed that the minor viral clones were promptly eliminated in immediate virologic responders, the originally-inhabited major viral clones persisted 1 week after peg-IFNα2b plus RBV administration. Thus, further analyses are required to clarify how viral heterogeneity might be associated with the response to anti-viral therapy.

DAAs are promising drugs that could be more effective than peg-IFNα plus RBV therapy [33]. These DAAs include HCV NS3/4A protease and NS5B RNA-dependent RNA polymerase inhibitors, both of which have currently advanced to phase 1-3 trials. Increasing evidence, however, has clearly revealed that monotherapy with DAAs poses a high risk for the selection of resistant variants because of the high genetic heterogeneity of HCV [20]. Several studies reported the low prevalence of DAAs resistant mutants as the dominant clones in treatment-naïve cases [21], [34]-[36]. For example, Kuntzen et al showed that drug-resistant mutations were detectable by conventional sequencing at individual frequencies between 0.3% and 2.8% in a treatment-naïve genotype 1 HCV-infected population [21]. In sharp contrast, ultra-deep sequencing identified that DAAs-resistant variants are common among treatment-naïve patients. Indeed, ultra-deep sequencing showed that 26 of 27 (96%) treatment-naive Japanese patients enrolled in this study possessed at least two clones resistant to DAAs, while 70.2% of the mutants presented as a very minor population (less than 1%) in each individual. It remains unclear whether these minor drug-resistant mutations have clinical significance, because the DAAs are not yet approved here in Japan. Recent *in vitro* findings, however, showed that minor but preexisting resistant mutants in HCV replicon cells were selected and expanded after DAAs therapy [37]. Lu et al revealed that M414T mutants preexisting at a frequency of 0.22% and 0.18% in the treatment-naïve replicon population rapidly increased upon treatment with DAAs in a dose-dependent manner, reaching frequencies of 25% and 60% after 4 days of treatment. These findings suggest that those preexisting minor mutants might cause resistance against DAAs through the selection of dominant mutations. Thus, the significance of low-abundance variants in treatment-naïve patients requires further exploration.

The present study raises two limitations of ultra-deep parallel sequencing technology in the analyses of viral quasispecies. First, because the massive parallel ultra-deep sequencing platform is based on multitudinous short reads, it is difficult to separately evaluate the association between nucleotide sites mapped to different viral genome regions in a single viral clone. Indeed, it is difficult to clarify the potential mutational linkage between different viral genomic regions because of the short read length of the shotgun sequencing approach. Second, it is difficult to accurately analyze highly polymorphic regions such as the HVR by ultra-deep sequencing, because mutation findings strongly depend on mapping to the reference genome sequences. Thus, utilization of both conventional and ultra-deep sequencing technology might be necessary to fully clarify the significance and clinical relevance of the prominent HCV genomic heterogeneity.

In summary, using ultra-deep sequencing technology, we clearly demonstrated the extremely large genetic complexity in the genotype1b HCV derived from chronically infected patients. Although there was no significant difference in the level of viral complexity between immediate virologic responders and non-responders at baseline, immediate virologic responders, but not non-responders, showed a rapid reduction in the viral sequence variability at an early phase of peg-IFNα2b plus RBV administration. We also showed that drug-resistant mutants were widely present in treatment-naïve HCV-infected patients, indicating a putative risk for the expansion of resistant clones to DAAs. Further studies with a large number of patients are needed to fully elucidate the significance of viral heterogeneity in the clinical outcome of patients receiving anti-viral therapy.

## Materials and Methods

### Patients

The participants comprised 27 Japanese adult chronic hepatitis patients with genotype 1b HCV infection and the mean baseline level of serum HCV RNA determined by TaqMan RT-PCR (Applied Biosystems, Foster City, CA) was 6.9 log IU/ml. All patients received conventional peg-IFNα2b plus RBV combination therapy (Schering-Plough, Kenilworth, NJ) at Kyoto University and affiliated hospitals from February 2007 to December 2008. Indications for IFN-based combination therapy included high serum values of alanine aminotransferase and positivity for serum HCV RNA. Patients were treated with peg-IFNα2b (1.5 µg/kg) once per week, combined with daily oral RBV for 48 weeks [38]. The RBV dose was 600 mg/day in patients weighing less than 60 kg, 800 mg/day in those weighing at least 60 kg but less than 80 kg, and 1000 mg/day in those weighing 80 kg or more.

In this study, immediate virologic responders were defined as patients whose serum HCV RNA levels declined by more than 2 log IU/mL after 1 week of treatment with peg-IFNα2b plus RBV, while non-responders were defined as those whose serum HCV RNA levels declined less than 2 log IU/mL after peg-IFNα2b plus RBV administration. Of the original 27 patients, the serum before and 1 week after initiating treatment with peg-IFNα2b plus RBV of 16 cases was available for further analyses, and 8 of these cases were defined as immediate virologic responders and 8 cases were defined as non-responders. Among these non-responder cases, the serum HCV RNA levels in 6 of 8 (75.0%) patients changed by less than 1 log IU/mL after 1 week of treatment. The decline in HCV RNA levels in the remaining 2 cases was slightly over 1 log IU/mL (1.2 and 1.4 log IU/mL).

The ethics committee at Kyoto University approved the studies, and written informed consent for participation in this study was obtained from all patients.

### Direct population Sanger sequencing

To define the representative reference sequences of full-length HCV in each clinical specimen, all samples were first subjected to direct population Sanger sequencing using Applied Biosystems 3500 Genetic Analyzer (Applied Biosystems, Foster City, CA) [39]. Serum samples were obtained before the start and at 1 week after initiation of peg-IFNα2b and RBV combination therapy. Total RNA was extracted from 140 µL of serum using a QIAamp Viral RNA Mini kit (QIAGEN, Valencia, CA) and reverse-transcribed in a volume of 20 µL with the One step RNA PCR Kit AMV (Takara Bio, Ohtsu, Japan).

HCV genomes were amplified using Phusion High-Fidelity DNA polymerase (FINZYMES, Espoo, Finland). Oligonucleotide primers were designed to amplify the first-half (∼5,000 bps) and the latter-half (∼4,500 bps) of the genotype 1b HCV genome sequences (Table S3).

PCR products purified by the QIAquick Gel Extraction kit (Qiagen) were assayed for direct sequencing [40]. Nucleotide sequences of PCR products were determined using an ABI Prism Big Dye Terminator Ready Reaction Kit (Applied Biosystems). The serum of a healthy volunteer was used as a negative control.

### Massive-parallel ultra-deep sequencing

Paired-end sequencing with multiplexed tags was carried out using the Illumina Genome Analyzer II. End-repair of DNA fragments, addition of adenine to the 3′ ends of DNA fragments, adaptor ligation, and PCR amplification by Illumina-paired end PCR primers were performed as described previously [41].

Briefly, the viral genome sequences were amplified with high-fidelity PCR and sheared by nebulization using 32 psi N2 for 8 min and the sheared fragments were purified and concentrated using QIAquick PCR purification Kit (Qiagen). The overhangs resulting from fragmentation were then converted into blunt ends using T4 DNA polymerase and Klenow enzymes, followed by the addition of terminal 3′ adenine-residues. Next, one of the adaptors containing six unique base pair (bp) tags, such as “ATCACG” and “CGATGT” (Multiplexing Sample Preparation Oligonucleotide Kit, Illumina), was ligated to each fragment using DNA ligase. Adaptor-ligated DNAs in the range of 200 to 350 bp were then size-selected by agarose gel electrophoresis. These libraries were amplified independently using a minimal PCR amplification step of 18 cycles with Phusion High-Fidelity DNA polymerase and then purified using a QIAquick PCR purification Kit for a downstream assay. Cluster generation and sequencing was performed for 64 cycles on the Illumina Genome Analyzer II following the manufacturer's instructions. Obtained images were analyzed and base-called using GA pipeline software version 1.4 with default settings provided by Illumina.

### Genome Analyzer sequence data analysis

Using the high performance alignment software “NextGene” (SoftGenetics, State College, PA), the 64 base tags obtained from the Genome Analyzer II reads were aligned to the reference HCV RNA sequences of ∼9200 bp that were determined by direct population Sanger sequencing in each clinical specimen. Entire reads were removed from the analysis when the median quality value score was below 20 and when containing more than 3 uncalled nucleotides. The low quality bases were trimmed from reads when more than 3 consecutive bases fell below a quality value score of 16. Based on the above criteria, reads with 90% or more bases matching a particular position of the reference sequence were aligned. Each position of the viral genome was assigned a coverage depth, representing the number of times the nucleotide position was sequenced.

### Statistical analysis

Results are expressed as mean or median values and range (minimum and maximum). Pretreatment values were compared using the Mann–Whitney U-test. Categorical variables were analyzed by Fisher's exact test. *P* values of less than 0.05 were considered statistically significant. The viral quasispecies nature was evaluated by analyzing the genetic complexity based on the number of different sequences present in the population. Genetic complexity was determined by Shannon entropy values calculated as follows:
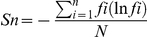
where *n* is the number of different species identified, *fi* is the observed frequency of the particular variant in the quasispecies, and *N* is the total number of clones analyzed [23], [42]. Statistical comparisons of complexity between two groups were made using the Wilcoxon rank sum test or the Mann–Whitney U-test.

## Supporting Information

Figure S1 **Relationship between serum HCV RNA levels and the number of resistant mutant.** No correlation was observed between serum HCV RNA levels (log IU/ml) and the number of resistant mutations against direct-acting antivirals in 27 cases in this study.
(TIF)

Table S1 Aligned reads, nucleotides, and mean coverage of each reference sequence in all patients.
(DOC)

Table S2 Mean genetic complexity in each viral genomic region of the 8 immediate virologic responders and 8 non-responders at pre-treatment and 1 week after IFN therapy.
(DOC)

Table S3 The oligonucleotide primers for PCR amplifying the whole HCV sequences.
(DOC)
