# Supplementary figures and images for: Genetic Heterogeneity of Hepatitis C Virus in Association with Antiviral Therapy Determined by Ultra-Deep Sequencing
Source: PLoS One. 2011 Sep 22;6(9):e24907. doi: 10.1371/journal.pone.0024907 (PMC3178558; doi:10.1371/journal.pone.0024907)

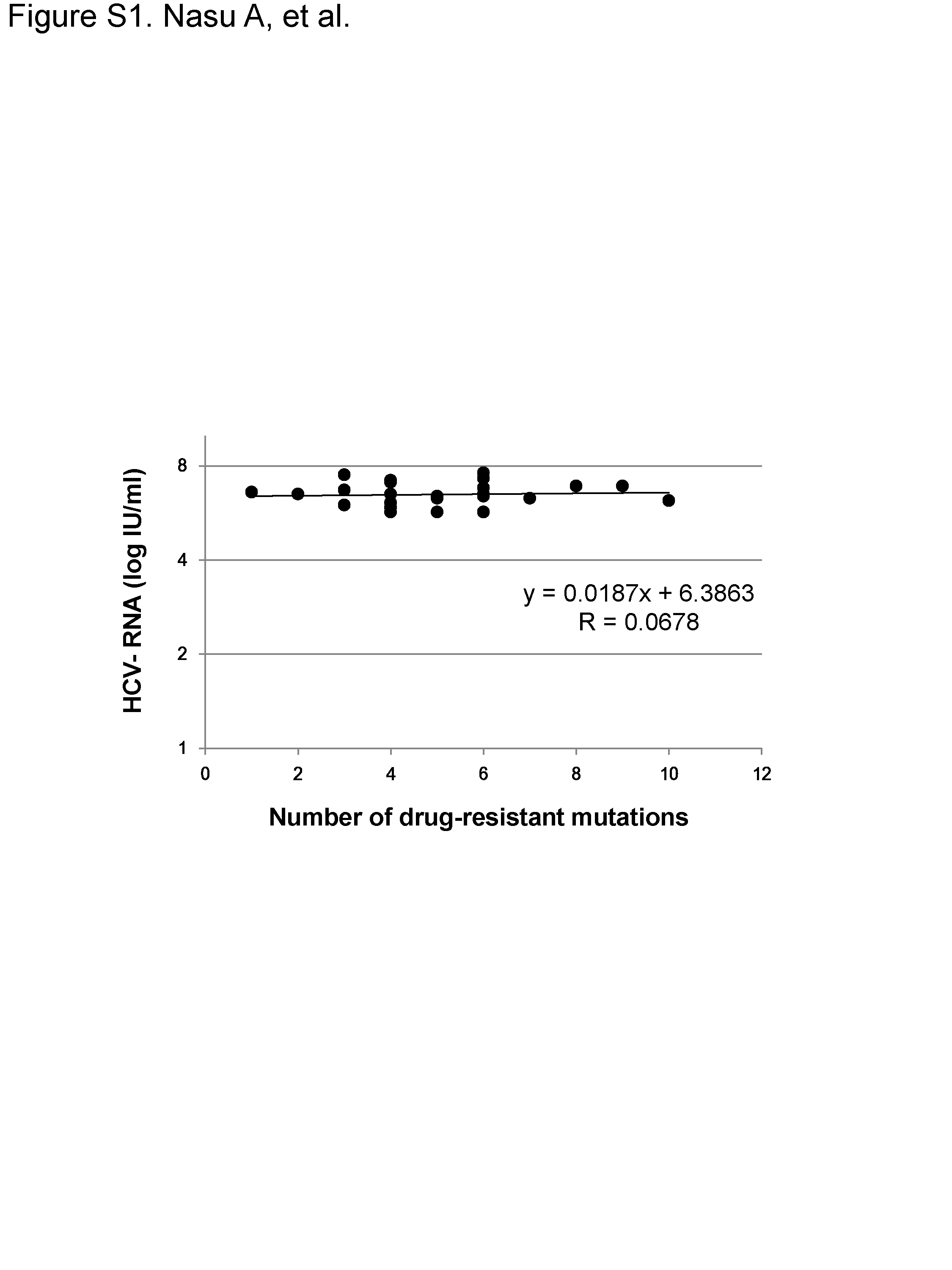

Supplement: Figure S1 — Relationship between serum HCV RNA levels and the number of resistant mutant. No correlation was observed between serum HCV RNA levels (log IU/ml) and the number of resistant mutations against direct-acting antivirals in 27 cases in this study. (TIF) [file pone.0024907.s001.tif]
