# Supplementary material for: Genetic Heterogeneity of Hepatitis C Virus in Association with Antiviral Therapy Determined by Ultra-Deep Sequencing
Source: PLoS One. 2011 Sep 22;6(9):e24907. doi: 10.1371/journal.pone.0024907 (PMC3178558; doi:10.1371/journal.pone.0024907)
Supplement: Table S1 — Aligned reads, nucleotides, and mean coverage of each reference sequence in all patients. (DOC) [file pone.0024907.s002.doc]

**Table S1. Aligned reads, nucleotides, and mean coverage of each reference sequence in all patients**

|  | **Pre-treatment** | | | **1week after IFN therapy** | | |
| --- | --- | --- | --- | --- | --- | --- |
|  | **Aligned reads** | **Aligned nucleotides** | **Mean coverage** | **Aligned reads** | **Aligned nucleotides** | **Mean coverage** |
| **Immediate virologic responder** | | |  |  |  |  |
| Patient #1 | 203,738 | 12,771,465 | 1440.6 | 103,087 | 6,458,834 | 720.5 |
| #2 | 213,501 | 13,403,768 | 1498.8 | 324,563 | 20,375,731 | 2278.4 |
| #3 | 252,976 | 15,875,343 | 1765.3 | 183,103 | 11,510,141 | 1279.9 |
| #4 | 218,790 | 14,126,650 | 1506.2 | 160,104 | 10,340,348 | 1102.5 |
| #5 | 342,251 | 22,091,819 | 2346.2 | 158,473 | 10,230,484 | 1086.5 |
| #6 | 215,293 | 13,461,679 | 1435.3 | 242,767 | 15,165,843 | 1617 |
| #7 | 154,823 | 9,656,618 | 1029.6 | 75,387 | 4,730,674 | 504.4 |
| #8 | 506,240 | 31,670,069 | 3376.7 | 1,608,219 | 100,692,944 | 10,736 |
| **Non-responder** | | |  |  |  |  |
| Patient #9 | 229,759 | 14,835,512 | 1580.6 | 392,106 | 25329060 | 2698.6 |
| #10 | 397,536 | 25,697,615 | 2719.9 | 291,057 | 18810023 | 1990.9 |
| #11 | 310,272 | 19,373,262 | 2065.6 | 438,908 | 27394183 | 2920.8 |
| #12 | 134,215 | 8,674,825 | 924.9 | 350,220 | 22101614 | 2356.5 |
| #13 | 217,764 | 13,556,407 | 1445.4 | 299,092 | 18666086 | 1990.2 |
| #14 | 314,207 | 19,696,768 | 2198.3 | 312,060 | 19565056 | 2183.6 |
| #15 | 103,453 | 6,485,932 | 896.6 | 182,707 | 11449252 | 1582.7 |
| #16 | 345,715 | 21,670,237 | 2406.2 | 569,034 | 35724100 | 3966.7 |
