# Supplementary material for: Genetic Heterogeneity of Hepatitis C Virus in Association with Antiviral Therapy Determined by Ultra-Deep Sequencing
Source: PLoS One. 2011 Sep 22;6(9):e24907. doi: 10.1371/journal.pone.0024907 (PMC3178558; doi:10.1371/journal.pone.0024907)
Supplement: Table S2 — Mean genetic complexity in each viral genomic region of the 8 immediate virologic responders and 8 non-responders at pre-treatment and 1 week after IFN therapy. (DOC) [file pone.0024907.s003.doc]

**Table S2. Mean genetic complexity in each viral genomic region of the 8 immediate virologic responders and 8 non-responders at pre-treatment and 1 week after IFN therapy**

|  | **Immediate virologic responders (N=8)** | | **Non-responders (N=8)** | |
| --- | --- | --- | --- | --- |
| **Viral genomic region** | **Pre-treatment** | **1 week after IFN therapy** | **Pre-treatment** | **1 week after IFN therapy** |
| core | 0.049202 | 0.037143 | 0.057212 | 0.049223 |
| E1 | 0.084084 | 0.040629 | 0.073774 | 0.063830 |
| E2 | 0.138622 | 0.085330 | 0.083368 | 0.082092 |
| p7 | 0.101334 | 0.047573 | 0.089429 | 0.077217 |
| NS2 | 0.094884 | 0.046611 | 0.090612 | 0.076465 |
| NS3 | 0.066464 | 0.046430 | 0.075507 | 0.063272 |
| NS4A | 0.080041 | 0.053932 | 0.083677 | 0.070757 |
| NS4B | 0.071097 | 0.051226 | 0.077480 | 0.064993 |
| NS5A | 0.079645 | 0.055380 | 0.079915 | 0.068606 |
| NS5B | 0.063151 | 0.048397 | 0.064880 | 0.059783 |
