# Supplementary material for: Genetic Heterogeneity of Hepatitis C Virus in Association with Antiviral Therapy Determined by Ultra-Deep Sequencing
Source: PLoS One. 2011 Sep 22;6(9):e24907. doi: 10.1371/journal.pone.0024907 (PMC3178558; doi:10.1371/journal.pone.0024907)
Supplement: Table S3 — The oligonucleotide primers for PCR amplifying the whole HCV sequences. (DOC) [file pone.0024907.s004.doc]

**Table S3. The oligonucleotide primers for PCR amplifying the whole HCV sequences**

| **Primer name** | **5'-Position*** | **Sequence** | **Forward / Reverse** | **Usage** |
| --- | --- | --- | --- | --- |
| Whole _Fw | 12 | GCCAGCCCCCTGATGGGGGCGACACTCCAC | Forward | RT and 1^st^ round PCR |
| Whole_Rv | 9405 | GCCTATTGGCCTGGAGTGTTTAGCTC | Reverse | RT and 1^st^ round PCR |
| Amp1_Fw | 47 | TGTCTTCACGCAGAAAGCGTCTAG | Forward | 2^nd^ round PCR for amplicon 1 |
| Amp1_Rv | 5305 | GCTTAAGTGACGACCTCCAGGTCAGCCGACAT | Reverse | 2^nd^ round PCR for amplicon 1 |
| Amp2_Fw | 5017 | CAGGCCTCACCCACATAGAT | Forward | 2^nd^ round PCR for amplicon 2 |
| Amp2_Rv | 9290 | TCGGGCACGAGACAGGCTGTGATATATGTCT | Reverse | 2^nd^ round PCR for amplicon 2 |

*All positions are relative to HCV genotype 1b sequence HCV-J (GenBank D90208)
